# Allele Frequency Changes Provide Evidence for Selection and Identification of Candidate Loci for Survival in Red Clover (*Trifolium pratense* L.)

**DOI:** 10.3389/fpls.2019.00718

**Published:** 2019-06-11

**Authors:** Åshild Ergon, Leif Skøt, Vegard Eriksen Sæther, Odd Arne Rognli

**Affiliations:** ^1^Department of Plant Sciences, Faculty of Biosciences, Norwegian University of Life Sciences, Ås, Norway; ^2^Institute of Biological, Environmental and Rural Sciences, Aberystwyth University, Aberystwyth, United Kingdom

**Keywords:** establishment success, genetic shift, population genomics, SNP, GBS, *F*_ST_, persistence, pool

## Abstract

Survivor populations of red clover (*Trifolium pratense* L.) from plots in a field experiment in southern Norway were genetically characterized using genotyping by sequencing, and compared with the original population and each other. Genetic differentiation between populations was characterized on the basis of allele frequencies of single nucleotide polymorphisms (SNPs), using principal component analysis. SNPs that had been under selection, i.e., SNPs with significantly different allele frequencies in survivor populations relative to the original population, or between survivor populations that had received different treatments, were identified by analysis of *F*_ST_ values, using BayeScan and a simple and stringent *F*_ST_-based test utilizing replicate populations from the field experiment. In addition, we tested the possibility of pooling DNA samples prior to sequencing, and pooling leaf samples prior to DNA extraction and sequencing, followed by allele frequency estimation on the basis of number of variant reads. Overall, survivor populations were more different from each other than from the original population, indicating random changes in allele frequency, selection in response to local variation in conditions between plots in the field experiment, or sampling error. However, some differentiation was observed between plots sown as pure stands or species mixtures, plots sown at different densities, and plots subjected to different harvesting regimes. Allele frequencies could be accurately estimated from pooled DNA, and SNPs under selection could be identified when leaf samples were pooled prior to DNA extraction. However, substantial sampling error required replicate populations and/or a high number of sampled individuals. We identified a number of chromosomal loci that had been under selection in pure stand plots relative to the original sown population, and loci that had been under differential selection in pure stands of red clover vs. red clover grown in species mixtures. These are all candidate loci for establishment success or persistence in red clover.

## Introduction

Red clover (*Trifolium pratense* L.) is a perennial legume used extensively in production of silage and hay in temperate regions, where it is cultivated in mixture with grasses such as timothy, meadow fescue and perennial ryegrass. It has high yields, can largely replace nitrogen fertilization through its symbiosis with nitrogen-fixing rhizobia, and it contributes to protein-rich forage and high intake (Frame et al., 1998; Abberton and Marshall, 2005). However, although red clover is a perennial, its persistence is limited, and it is often the species that first disappears from a species mixture (Ms). Improving persistence is therefore, a major breeding goal in most breeding programs (Taylor, 2008; Boller et al., 2010). Red clover is an outbreeding species with a gametophytic self-incompatibility system (Taylor, 1982), and thus there is a considerable amount of genetic variation within cultivars, which are usually synthetic populations with a large number of parents. Red clover has a genome size of 420 Mb (Sato et al., 2005). Sequences of the red clover genome have been published (Ištvánek et al., 2014; De Vega et al., 2015), but only the latter of which represents a draft genome at pseudomolecule level, covering 309 Mb of the genome. This facilitates particularly the identification of genomic regions potentially under selection.

Persistence is a complex trait controlled by many different genetic and environmental factors. It may be defined purely as survival over years, or alternatively, as maintenance of annual yield over years. These two measures can be correlated (e.g., Herrmann et al., 2008), but there may also be negative associations between harvestable biomass produced and survival during a subsequent stressful period, such as a winter (Therrien and Smith, 1960; Ergon, 2017). Biotic and abiotic stresses such as fungal pathogens, nematodes and insect herbivores, abiotic stresses, and competition from grasses under high N fertilization levels, are factors that can cause reduced persistence of red clover (Lager and Gerhardson, 2002; Abberton and Marshall, 2005; Taylor, 2008; Boller et al., 2010; Annicchiarico et al., 2015). Cutting frequency has been found to be of lesser importance (Coulman and Kielly, 1988; Wiersma et al., 1998). Positive correlations have been found between persistence and stem height or leaf size (Herrmann et al., 2008) and adventitious root formation (Montpetit and Coulman, 1991a,b). The ability to regrow after repeated defoliation is likely to be related to presence of leaves low in the canopy, level of root energy reserves and number of crown buds, as indicated for lucerne (Brummer and Bouton, 1991, 1992). Little is known about the genetic control of persistence, but it is likely that interaction with environmental factors are important. To our knowledge only one report on QTLs for persistence (Herrmann et al., 2008), and very few reports on QTLs for related traits such as winter survival and disease resistance (Klimenko et al., 2010) and vigor (Herrmann et al., 2008) are published.

In agricultural fields in Norway, seed mixtures, often containing around 10% (weight) of red clover, are commonly sown at a rate of 20–30 kg ha^-1^. With a 1000 seed weight of 2 g (diploid red clover), this equates to a red clover seeding rate of around 1 × 10^6^ – 1.5 × 10^6^ seeds ha^-1^. Only a fraction of the sown plants will survive the first years due to competition and stress. For example, in pure red clover stands sown at a rate of 18 kg ha^-1^, 40, 27 and 18% of the number of plants expected to germinate according to a germination test, had survived by the end of the first (establishment year), second and third growing season, respectively (Marley et al., 2003). However, the initial survival rate is likely higher in species mixtures because intraspecific competition is usually higher than interspecific competition.

Natural selection can guide breeding (Henry and Nevo, 2014), particularly when it comes to a trait like the survival component of persistence. Selection of high persistence in breeding programs is usually done by selecting plants that have survived under field conditions for around 3 years. Such survivor populations of both red and white clover have been found to have experienced a shift in the genetic composition of the populations relative to the original populations, measured either with molecular markers or with phenotyping of offspring (Annicchiarico and Piano, 1997; Collins et al., 2001, 2002, 2012; Dalmannsdóttir et al., 2001; Frankow-Lindberg, 2001; Helgadóttir et al., 2001; Göransson et al., 2012; Ergon and Bakken, 2016). In white clover grown under Nordic conditions, such shifts have been associated with improved winter survival or related traits (Dalmannsdóttir et al., 2001; Frankow-Lindberg, 2001; Göransson et al., 2012).

In this paper, we utilized survivor populations to investigate whether non-random selection could be detected within one generation of red clover growing in the field for 2.5 years, and to what extent any such selection had acted on the population structure or on individual loci. We first aimed to identify loci controlling persistence by detecting loci with significantly altered allele frequencies in survivor populations compared to the originally sown population (study 1). We based our analyses on single nucleotide polymorphism (SNP) data obtained from genotyping by sequencing (GBS) of individuals from the original population and survivor populations. We then tested whether reliable allele frequencies could be obtained by sequencing pools of individual DNA samples rather than sequencing the individual samples themselves (study 2). Finally, we used GBS-derived SNP data from pools of leaf samples to investigate whether different loci had been selected in red clover survivors grown in pure stands as compared to red clover survivors grown in species mixtures (study 3).

## Materials and Methods

### Plant Material and Genotyping

The diploid red clover cultivar “Lea” (Graminor, Norway) was included in a larger field experiment, sown in two replicates at Ås, Norway, in June 2010 (Ergon et al., 2016, Supplementary Figure S1). The plot size was 7.5 m^2^, seeds were sown at a total seed rate of 10 (low) or 20 (high) kg ha^-1^, either as red clover pure stand (Ps) or as mixed stands (Ms) sown with equal amounts (seed weight) of red clover, white clover, perennial ryegrass and tall fescue. With an approximate thousand seed weight of diploid red clover of 2 g, this equals approximately 3750 or 7500 seeds per Ps plot, and 938 or 1875 seeds per Ms plot. Plots had been harvested either 3 or 5 times a year (3H and 5H). Leaf blades were sampled from survivor populations (i.e., plots) in October 2012, and stored at -80°C.

In 2013, DNA was extracted from leaves of 48 or 47 survivor plants randomly selected from Ps survivor populations sown at high seeding rate and harvested 3 or 5 times a year (two plots from each harvesting regime, sample set 1, Table 1), and from leaf samples of 88 individuals of the original population seeded in the greenhouse (sample set 2), using DNeasy 96 Plant Kit (Qiagen). GBS libraries were made for each of the 278 individuals. In order to test how well allele frequencies can be estimated from DNA pools, equal amounts of DNA from the 88 individuals of the original population were pooled and distributed among seven tubes from which 7 replicate GBS libraries were made (sample set 3). GBS library preparation and sequencing, as well as SNP calling, was done at the Institute for Genomic Diversity, Cornell University, according to Elshire et al. (2011). The enzyme ApeK1 was used for digestion of genomic DNA, and the GBS UNEAK analysis pipeline, an extension to the Java program TASSEL (Bradbury et al., 2007), was used to call bi-allelic SNPs from the sequenced GBS libraries.

**Table 1:** Overview of genotyped material.

Sample set	Populations	Number of sampled individuals per population	Type of DNA samples	Round of GBS and SNP calling	Comparison of allele frequencies
1	Four survivor populations^1^	47–48	DNA from separate individuals	1	Study 1: Survivor populations vs. original population
2	Original population^2^	88	DNA from separate individuals	1	Study 1: Survivor populations vs. original population Study 2: Individuals vs. DNA pool
3	Original population	88	Pools of all DNA samples in set 2^3^	1	Study 2: Individuals vs. DNA pool
4	Eight survivor populations, sown at low or high seeding density and in pure stand or in mixture with grasses^4^	100^5^	Leaf samples pooled prior to DNA extraction	2	Study 3: Pure stands vs. mixed stands and high seeding density vs. low seeding density


*Each experimental plot represents one survivor population. Leaves for sample set 1 and 4 were taken from surviving individuals in experimental plots in a field experiment (Ergon et al., 2016) in October 2012. Samples in set 2 was taken from individuals from the original seed bag, sown and grown in a greenhouse. ^*1*^All sown at low seeding rate and in pure stand; two populations had been harvested 3 times a year, while the other two had been harvested 5 times a year. ^*2*^Sown and cultivated in a greenhouse prior to sampling.^*3*^Seven replicate pools were made, each consisting of equal amount of DNA from each of the 88 individuals. ^*4*^All harvested 3 times a year; two populations from each of the four possible combinations of seeding density and stand type. ^*5*^For two of the populations (those sown at high seeding rate and in pure stand) there were 3 replicate samples, each made up of leaves from 100 individuals.*

In 2017, DNA was also extracted from leaf samples of some of the other survivor populations that had been kept at -80°C. This time, DNA was extracted from pools of leaves (one leaf from each of 100 random individuals from each population, sample set 4, Table 1), using DNeasy Plant Maxi Kit (Qiagen). Leaves were sampled from four red clover Ps survivor populations, and four Ms survivor populations, all which had been harvested 3 times a year. Two plots of each stand type had been sown at high seeding rate (H) and two at low seeding rate (L). From the Ps H populations, three replicate samples, each consisting of one leaf from each of 100 random individuals, were sampled in order to evaluate the reproducibility of the sampling. GBS library preparation and sequencing, as well as SNP calling for the resulting 12 samples, was done at Beijing Genomic Institute, using ApeK1 as the restriction enzyme. Sequences were aligned to the red clover genome using SOAP (Li et al., 2009b), and bi-allelic SNPs were detected with SOAPsnp (Li et al., 2009a).

### Analysis of Genotype Data

#### Study 1: Genetic Changes in Survivor Populations as Compared to the Originally Sown Population, Based on Genotyping of Individuals

For the libraries made from individuals in sample set 1 and 2 (Table 1), a minimum of ten reads in total, and, in the case of heterozygotes, a minimum of two reads for each of the alleles, was required to maintain the genotype for each genotype and SNP combination. For analysis of the changes in survivor populations (sample set 1) as compared to the original population (sample set 2), SNPs with an established genotype for a minimum of 25 individuals in all five populations, and minor allele frequency (MAF) > 0.05 in the original population, were used (4966 SNPs).

Principal component analyses (PCAs) were performed in The Unscrambler X v.10.3 (Camo Software, Norway) in order to visualize differentiation between populations as well as population structure. For the visualization of population differentiation MAF of each SNP in each population were used as input data. For visualization of population structure the genotype of each SNP and all individuals were used as input data.

In order to identify SNP loci that had been under selection two different methods were used. With the first method, a simple *F*_ST_-based method, pairwise *F*_ST_-values (original population vs. each of the four survivor populations) were calculated for each SNP and pair of populations as $\frac{\bar{q^{2}}-{\bar{q}}^{2}}{\bar{q}\left(1-\bar{q}\right)}$, where *q* is the allele frequency of a SNP variant and all the averages are calculated over the two populations compared. Secondly, a chi-square test was used to identify SNPs with significant *F*_ST_’s at different significance levels, using the test statistic *X^2^ = 2NF_ST_*, where *2N* = the sum of genotyped gametes in the two populations (Hedrick, 2011). Thirdly, SNPs with significant *F*_ST_ in all four survivor populations relative to the original population, were identified. Only these SNPs were regarded as having different allele frequency in survivor populations as compared to the original population. In order to control the high rate of false positives that can occur in multiple testing, corresponding overall estimates of the false discovery rate (FDR) were calculated for each significance level as $\frac{l^{*}P^{n}}{d}$, where *l* = number of SNP loci tested (4966), *P* = the significance level of the individual chi-square tests, *n* = the number of survivor populations tested against the original population (4), and *d* = the number of SNP loci identified with a significant *F*_ST_ in all n population pairs. For each SNP with a significant *F*_ST_ in all four survivor populations relative to the original population, the significance of the difference between the allele frequency in the original population and the combined population of 190 genotyped survivors (all four plots) was confirmed with Fishers exact test, using the tool at http://lh3lh3.users.sourceforge.net/fisher.shtml. The 64 bp tag sequences containing significant SNPs were blasted against the red clover draft genome (De Vega et al., 2015) at https://legumeinfo.org/, in order to identify map locations (defined as the best hit with at least 57 bp aligned and a maximum *e*-value of 1 e^-18^) and surrounding candidate genes.

Single nucleotide polymorphism outliers were also detected with BayeScan v 2.1 (Foll and Gaggiotti, 2008), using default input parameters. The allele frequency dataset described above was converted to allele numbers using the number of haploid genomes that had been genotyped for each population. BayeScan uses logistic regression to decompose locus-population *F*_ST_-values into population-specific and SNP-specific components. Population-specific *F*_ST_-values are based on the comparison between each population and the pool of all populations in the analysis. Outlier SNPs are identified as those, where the SNP-specific component is necessary to explain the observed variation. We ran the analysis with five defined populations (four survivor populations and the original population) and three defined populations (data from 3H populations pooled, data from 5H populations pooled, original population).

#### Study 2: The Use of Pooled Samples to Predict Allele Frequencies in Populations

For the seven libraries made from pools of individual DNA samples (sample set 3, Table 1), the sequence reads were filtered for minimum 10 and maximum 126 reads per SNP and replicate library. Reads were subsequently pooled across libraries, and allele frequencies were calculated based on number of reads. For comparison of allele frequencies obtained from GBS of individuals (sample set 2) vs. pooled DNA (sample set 3), SNPs with an established genotype for a minimum of 25 individuals and MAF > 0.05 among individuals in the original population, were used (8218 SNPs).

#### Study 3: Differential Selection in Pure Stands vs. Mixed Stands, Based on Genotyping of Pooled Tissue Samples

Sequencing of libraries made from the DNA extracted from leaf pools in sample set 4 (Table 1) generated 8294 SNPs with 100–499 reads and MAF > 0.05 within all 12 samples. We discarded those that mapped to scaffolds not yet assigned a chromosomal locations, leaving 4556 SNPs for analysis. Allele frequencies were estimated on the basis of the number of reads. Differentiation between populations and replicate samples was analyzed with PCA as in experiment 1.

In order to identify SNPs potentially differentially selected in plots receiving different treatments, we used the same two methods as in study 1. For the simple *F*_ST_-based method, pairwise *F*_ST_ values between each of the four Ps populations and the average of the Ms populations, and between each of the four Ms populations and the average of the Ps populations, were calculated for each SNP. For the two plots with replicate samples, the average allele frequencies for each plot were used. Allele frequency differences were tested for significance as described in study 1. For a SNP locus to be identified as having been under differential selection, it was required that all eight *F*_ST_ values were significant. Estimates of FDR were calculated as in study 1, with *l* = 4556 and *n* = 4, because there are only four independent *F*_ST_ values. Fishers exact test was not performed due to the lack of individual genotypes.

BayeScan compares each population with all the other populations in the analysis. An identified SNP outlier may therefore not necessarily vary consistently between, e.g., all Ps and Ms populations. We therefore tested the effect of stand type, seeding density and the interaction for each significant SNP with a two-way analysis of variance using the GLM procedure in SAS Enterprise Guide v. 6.1, in order to identify SNPs that had significantly different allele frequencies in either different stand types or in different seeding densities. We also ran a BayeScan analysis in which the data from each of the eight populations were combined in two main groups, Ps and Ms.

## Results

### Study 1: Genetic Changes in Survivor Populations as Compared to the Originally Sown Population, Based on Genotyping of Individuals

**FIGURE 1:**
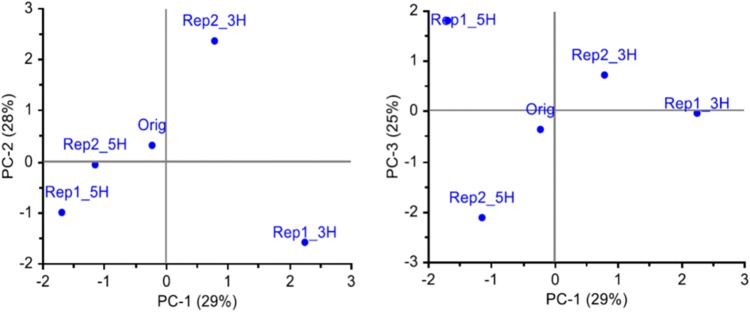
Genetic differentiation between the original population (Orig, 88 individuals) and four survivor populations sampled from four plots (47–48 individuals per population) in a field experiment 2.5 years after sowing, i.e., all plants are in the same generation (study 1). The survivor populations had been harvested three (3H) or five (5H) times a year, and there were two replicate plots of each harvesting regime. All SNPs that were successfully genotyped for a minimum of 25 individuals from each population, and had a MAF > 0.05 in the original population, were included (4966 SNPs). The three first principal components explaining the variation in allele frequencies are shown.

In study 1, the set of SNPs with at least 25 genotyped individuals in each of the five population samples and MAF > 0.05 in the original population (4966 SNPs), were used to characterize the genetic changes that had occurred in the field over the two and a half years. A PCA of MAF for each SNP and population showed that along the two first axes, the survivor populations had diverged from the original population in different directions (Figure 1). The two populations from the 5H harvesting regime were more similar to each other than the two populations from the 3H harvesting regime. The first axis, explaining 29% of the variation, separated the 3H populations from the 5H populations. The second axis (28% of the variation) separated the two 3H populations, and the third axis (25% of the variation) separated the two 5H populations. These results suggest that the survivor populations had diverged from the original population in different directions, and that most of the observed difference in allele frequencies was random.

**FIGURE 2:**
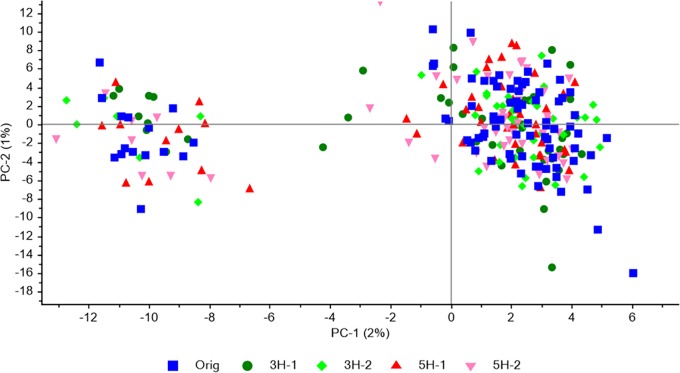
Population structure revealed by PCA of SNP genotype data for 88 individuals from the original population together with 47 or 48 individuals from each of the four survivor populations in study 1. All SNPs that were successfully genotyped in a minimum of 25 individuals from each population, and had a MAF > 0.05 in the original population, were included (4966 SNPs).

Population structure was analyzed with PCA of all individuals and the 4966 SNPs. The same weak structure observed in the original population, with one major and one minor subpopulation, was observed in each of four survivor populations (Figure 2). The two first principal components only explained 2 and 1% of the variation, respectively. The proportion of individuals in the minor cluster were 0.17 and 0.15 – 0.23 for the original population and the four survivor populations, respectively, indicating that the survival/mortality that had occurred in the field had not favored one subpopulation over the other.

**FIGURE 3:**
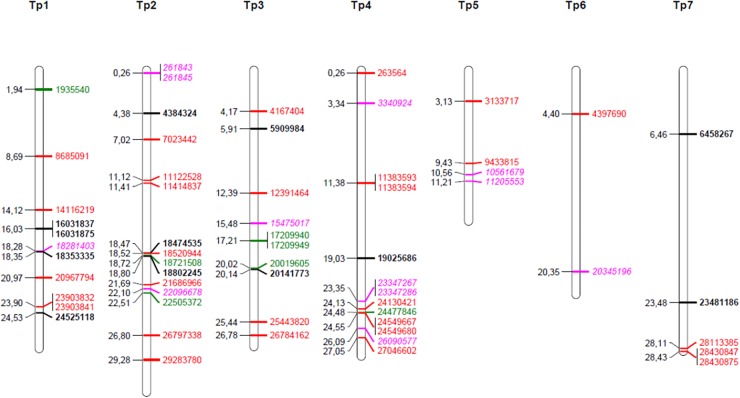
Map positions of chromosomal SNPs found to have been under selection. Black bold; selected in four survivor populations (pure stand, sown at high density) as compared to the original, sown population (study 1), red; differentially selected in survival populations from pure stand as compared to survival populations from species mixtures (study 3), green; differentially selected in populations as compared to populations sown at low seeding density (study 3), pink italic, selection affected by both stand type and seeding density (study 3).

The 4966 SNP loci were first screened for significant shifts in allele frequency between the original population and survivor populations with a simple test using *F*_ST_ values and a chi square test combined with FDR. When testing individual survivor populations separately, or when combining all survivors in one common population, significant FDR values could not be obtained; they were in the range of 0.4 – 0.8. Thus, the number of SNP loci identified was only about twice of what could be expected from chance alone. However, it is very unlikely that a locus has a shift in allele frequency due to chance alone in all four survivor populations. We therefore looked for SNP loci that had a significant shift in allele frequency in all four populations, and calculated a modified FDR accounting for this reduced number of expected false positives (see section “Materials and Methods”). Twenty-seven SNPs had a shift in allele frequency at *P* < 0.1 in all four survivor populations with an overall corresponding FDR of 0.018. Thirteen of these were also significant at *P* < 0.05 in all survivor populations with an overall FDR of 0.002 (Supplementary Table S1). The average shifts in allele frequency of the 27 SNPs across the four survivor populations ranged from 0.09 to 0.22 and the average *F*_ST_ values ranged from 0.017 to 0.061 (Table 2). For comparison, the *F*_ST_ value averaged across survivor populations and *all* 4966 loci was 0.005. When we tested the allele frequency difference of the 27 SNPs between all 190 survivors and the original population using Fishers exact test, all SNPs were significant at *P* < 0.01 - 0.000008 (Table 2). Sequence tags of 20 of the 27 SNPs could be mapped onto the red clover genome. Fourteen SNPs mapped to chromosomes (between two and four SNPs on each of chromosome 1, 2, 3, 4, and 7) (Table 2 and Figure 3), and six mapped to scaffolds not yet assigned to chromosomes (Supplementary Table S2).

**Table 2:** Sequence tags containing SNPs with a significant shift in allele frequency in four red clover pure stand survivor populations relative to the originally sown population (study 1), identified using a simple *F*_ST_-based method.

Sequence tag	Chromosomal position^1^	MAF^2^ in the original population (2N^3^)	Average allele frequency^4^ in survivor populations ± S.E. (2N)	Absolute average change in allele frequency	Average *F*_ST_ relative to the original population ± S.E. (significance level^5^)	Significance (P) in Fishers exact test^6^
TP24824	Tp3_5909984	0.46 (78)	0.68 ± 0.01 (54–70)	0.22	0.049 0.006 (**)	0.0008
TP31934		0.35 (104)	0.14 ± 0.02 (58–76)	0.21	0.061 0.014 (**)	0.00002
TP47052		0.35 (158)	0.55 ± 0.05 (70–90)	0.19	0.044 0.020 (*)	0.0001
TP107244		0.09 (76)	0.28 ± 0.03 (52–68)	0.19	0.060 0.013 (**)	0.0004
TP12160		0.29 (130)	0.47 ± 0.01 (48–64)	0.18	0.035 0.004 (**)	0.0008
TP6077		0.11 (84)	0.28 ± 0.03 (50–66)	0.17	0.048 0.013 (**)	0.001
TP21588	Tp1_16031837^§^	0.05 (138)	0.21 ± 0.03 (70–76)	0.16	0.058 0.015 (**)	0.000008
TP31385		0.36 (134)	0.21 ± 0.02 (60–70)	0.15	0.029 0.006 (*)	0.002
TP103112	Tp1_16031875_§_	0.05 (142)	0.19 ± 0.03 (68–80)	0.14	0.050 0.013 (**)	0.00002
TP13675		0.17 (156)	0.31 ± 0.01 (56–74)	0.14	0.027 0.003 (**)	0.002
TP129825		0.36 (154)	0.23 ± 0.01 (70–86)	0.14	0.023 0.004 (**)	0.003
TP110520		0.24 (110)	0.10 ± 0.01 (50–60)	0.14	0.034 0.004 (**)	0.002
TP112120		0.49 (174)	0.35 ± 0.01 (76–84)	0.13	0.019 0.003 (*)	0.005
TP81268	Tp7_6458267	0.46 (170)	0.60 ± 0.01 (80–92)	0.13	0.017 0.003 (*)	0.006
TP9013	Tp2_18802245	0.47 (162)	0.34 ± 0.01 (78–88)	0.13	0.018 0.004 (*)	0.006
TP98237	Tp4_20141773	0.08 (130)	0.21 ± 0.02 (50–74)	0.13	0.034 0.008 (*)	0.001
TP17472	Tp4_19025686	0.17 (112)	0.05 ± 0.01 (56–66)	0.12	0.036 0.005 (**)	0.0005
TP114603		0.09 (110)	0.20 ± 0.01 (56–76)	0.11	0.024 0.004 (*)	0.01
TP73363	Tp1_18353335	0.15 (128)	0.04 ± 0.01 (64–68)	0.11	0.036 0.010 (*)	0.0003
TP146594	Tp1_24525118	0.14 (108)	0.04 ± 0.01 (60–76)	0.10	0.034 0.007 (**)	0.0009
TP24591		0.19 (162)	0.09 ± 0.01 (70–84)	0.10	0.022 0.004 (*)	0.003
TP11471	Tp7_23481186	0.12 (172)	0.21 ± 0.01 (78–94)	0.10	0.018 0.005 (*)	0.007
TP32258		0.13 (128)	0.03 ± 0.00 (58–70)	0.10	0.034 0.004 (**)	0.0003
TP48637	Tp2_4384324	0.21 (168)	0.11 ± 0.01 (86–90)	0.10	0.017 0.002 (*)	0.005
TP88042		0.12 (140)	0.02 ± 0.01 (50–80)	0.10	0.037 0.011 (*)	0.0001
TP7660	Tp2_18474535	0.18 (170)	0.08 ± 0.01 (78–90)	0.09	0.022 0.008 (*)	0.003
TP120899		0.06 (136)	0.15 ± 0.01 (60–82)	0.09	0.023 0.005 (*)	0.009


^1^Pairs of SNPs located on the same sequence tag are labeled with §. ^*2*^MAF, minor allele frequency. ^*3*^2N, number of gametes genotyped. ^*4*^Frequency of the allele that was minor in the original population. ^5^*P < 0.1 for each of the four survivor populations (corresponding *FDR* = 0.018); **P < 0.05 for each of the four survivor populations (corresponding *FDR* = 0.002). ^*6*^Individuals from all four survivor populations were pooled and tested against the original population.

BayeScan did not detect any significant SNP outliers (FDR 0.05) when all five populations were defined, but when we combined the two 3H populations into one population, and the two 5H populations into another, in addition to the original population, one significant SNP was identified. This SNP, Tp3_16031875, was also identified with the simple *F*_ST_-based method (Table 2). We also attempted to identify SNP loci that had been under specific selection in either of the two harvesting regimes by looking for significant allele frequency differences between the 3H and the 5H survivor populations, using the simple *F*_ST_-based method. No such SNP loci could be detected, possibly due to the fact that there were only two replicate plots of each harvesting regime, and thus less power in the test.

### Study 2: The Use of Pooled Samples to Predict Allele Frequencies in Populations

**FIGURE 4:**
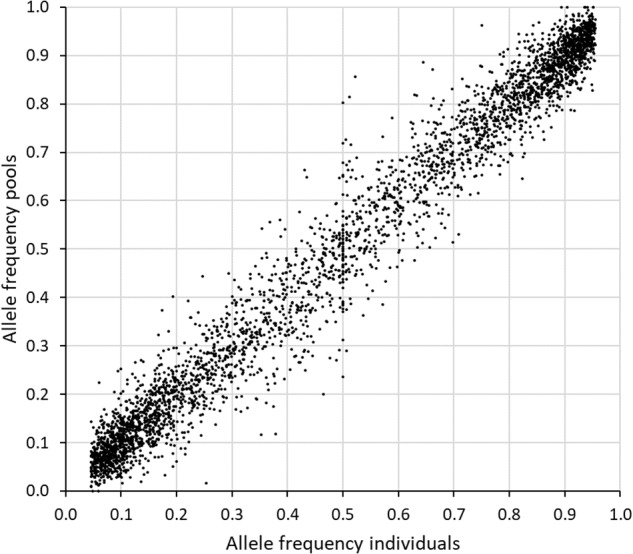
Comparison of the allele frequencies obtained when genotyping 88 individuals separately (*x*-axis) with those obtained when genotyping pools of equal amount of DNA from each individual (*y*-axis) in study 2. Seven GBS libraries were made from the pooled sample and the reads from these were combined prior to calculation of allele frequencies. The 3716 SNPs with an established genotype for at least 25 individuals, MAF > 0.05 among individuals, and 100–499 reads in the pooled sample, were included here.

A comparison was made between the allele frequencies obtained by genotyping individuals from the original population, with the allele frequency estimates obtained when sequencing a pool of equal amounts of DNA from each individual (sample set 2 and 3). The inaccuracy of the allele frequency estimates based on GBS of pooled DNA was higher for SNPs with less than 50 reads or more than 600 reads from the sequencing of the pool (Table 3). The best accuracy was obtained between 150 and 399 reads. When only SNPs in this range were included (2313 SNPs), the average deviation in allele frequency was 0.036 and the correlation 0.98. Expanding the range to 100–499 reads resulted in a much higher number of SNPs (3726), an average deviation of 0.039 and a correlation of 0.97 (Figure 4).

**Table 3:** Average deviation in allele frequency estimates obtained when genotyping pooled samples consisting of equal amounts of 88 individuals as compared to genotyping the individuals separately (study 1).

Number of reads	Number of SNPs	Average deviation in allele frequency	Correlation (r^2^)
10–49	2509	0.081	0.91
50–99	1885	0.050	0.96
100–149	1272	0.044	0.97
150–199	930	0.036	0.98
200–299	1009	0.036	0.97
300–399	374	0.036	0.98
400–499	141	0.044	0.97
500–599	74	0.064	0.94
>599	24	0.100	0.82


*SNPs with an established genotype for at least 25 individuals and MAF <0.05 among individuals were included, and grouped according to the sum of reads obtained from 7 pooled samples.*

### Study 3: Differential Selection in Pure Stands vs. Mixed Stands, Based on Genotyping of Pooled Tissue Samples

**FIGURE 5:**
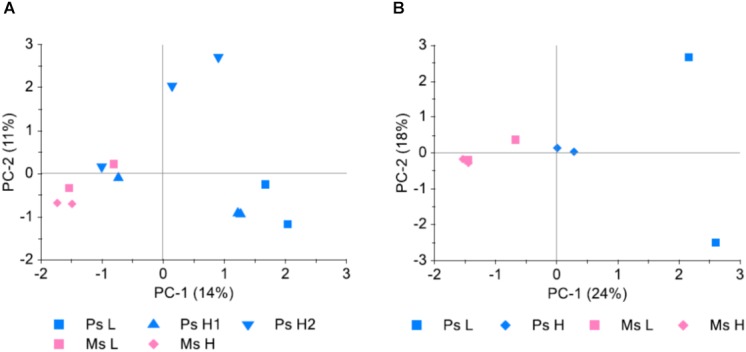
Genetic differentiation between survivor populations sampled from four pure stand plots (Ps, red clover only) and four mixed stand plots (Ms, red clover growing in mixture with white clover, perennial ryegrass and tall fescue), sown at high (H) or low (L) seeding density (study 3). Hundred leaves were collected from each plot and pooled prior to DNA extraction and GBS. For the Ps H plots (Ps H1 and Ps H2), three replicate samples, each consisting of 100 leaves, were sampled. SNPs which could be mapped to chromosomes and had 100–499 reads and MAF >0.05 were included (4556 SNPs). **(A)** Principal component analysis (PCA) of minor allele frequencies (MAF) in all 12 samples. **(B)** PCA of MAF in the 8 different populations. MAF for Ps H populations was the average of the three replicate samples.

Study 3 served both to investigate the possibility of pooling individual leaf samples prior to DNA extraction and GBS, and to investigate the possible differential selection that had occurred in survivor populations as a result of different stand types and seeding densities. A comparison was made between allele frequencies obtained from genotyping of DNA extracted from three replicate pools of leaf tissue from each of two Ps H populations, each pool consisting of 100 leaves. The average pairwise deviation in allele frequency between replicates was 0.056–0.057, while the average pairwise correlation of allele frequencies (r^2^) was 0.90 in both populations. Principal component analysis (PCA) (Figure 5A) revealed that the random variation between replicate samples from the same population was at least as large as the variation between samples from different populations. When allele frequencies were averaged across the three replicates this random variation appeared to be reduced (Figure 5B), indicating that a large part of the variation between samples was due to random variation which was reduced with the averaging of the three replicates.

The first principal component, explaining 24% of the variation in MAF between populations, separated Ms, Ps H, and Ps L populations (Figure 5B), suggesting differential selection as a result of the different treatments in the field experiment. The largest differences were between populations belonging to different stand types. There was also a difference between Ps populations sown at different seeding densities, possibly only detectable in Ps due to the higher number of individuals genotyped in Ps H populations.

The simple *F*_ST_-based method identified 11 SNPs at *P* < 0.1 (FDR 0.04), six SNPs at *P* < 0.05 (FDR 0.005) and one SNP at *P* < 0.01 (FDR 0.00005) (Figure 3, Table 4, and Supplementary Table S3). The difference in allele frequency between the average Ps population and the average Ms population for the 11 SNPs ranged from 0.17 to 0.37, and the average *F*_ST_-values ranged between 0.037 and 0.150, as opposed to a value of 0.005 across all 4556 SNPs. Thus, the differentiation between survival populations in this study was larger than the differentiation between the original population and survivor populations in study 1. BayeScan identified 156 SNPs with deviating allele frequency in at least one of the eight populations sampled (FDR 0.05). Testing each of these SNPs with analysis of variance showed that stand type had a significant effect on the allele frequency of 42 of these SNPs, while seeding density had an effect on 19, and 12 were affected by both factors (*P* < 0.05, Table 4). BayeScan analysis with only two defined populations – Ps and Ms – identified 59 significant SNPs. Thirty-three of these were among those 42 that had been identified as being affected by stand type. The 11 SNPs identified with the simple *F*_ST_-based method were also identified in both BayeScan analyses, and among those for which there was a significant effect of stand type.

**Table 4:** Single nucleotide polymorphisms (SNPs) with different allele frequencies in red clover populations 2.5 years after being sown in Ps orMs, and with low or high seeding density (study 3).

SNP^1^	Allele frequency difference		Significance of effects in analysis of variance^2^			Difference between stand types according to simple *F*_ST_-based test
			
	Stand types	Seeding densities	Stand type	Seeding density	Inter-action	
Tp1_1935540	0.04	0.13		*		
Tp1_8685091	0.11	0.03	*			
Tp1_14116219	0.13	0.01	*			
Tp1_18281403	0.12	0.13	*	**		
Tp1_20967794	0.17	0.05	**			
Tp1_23903832^§^	0.17	0.02	***		**	
Tp1_23903841^§^	0.19	0.02	***		**	
Tp1_27425320	0.14	0.01	*			*
Tp2_261843^§^	0.13	0.10	***	**		
Tp2_261845^§^	0.13	0.08	***	***		
Tp2_7023442	0.17	0.07	**			
Tp2_11122528	0.12	0.03	*			*
Tp2_11414837	0.14	0.02	*			
Tp2_18520944	0.19	0.04	**			
Tp2_18721508	0.06	0.15		*		**
Tp2_21686966	0.10	0.08	*			
Tp2_22096678	0.10	0.08	**	*		
Tp2_22505372	0.04	0.17		*		
Tp2_26797338	0.17	0.02	**			
Tp2_29283780	0.33	0.07	**			
Tp3_4167404	0.10	0.02	*			***
Tp3_12391464	0.16	0.14	*			
Tp3_15475017	0.11	0.14	*	*		
Tp3_17209940^§^	0.03	0.13		*		
Tp3_17209949^§^	0.03	0.13		*		
Tp3_20019605	0.05	0.10		*		
Tp3_25443820	0.09	0.07	*			
Tp3_26784162	0.14	0.07	*			
Tp4_263564	0.19	0.06	*			
Tp4_3340924	0.10	0.14	**	***		
Tp4_11383593^§^	0.36	0.09	**		*	
Tp4_11383594^§^	0.35	0.09	**		*	**
Tp4_23347267^§^	0.28	0.09	**	*		**
Tp4_23347286^§^	0.28	0.09	**	*		**
Tp4_24130421	0.13	0.05	*			**
Tp4_24477846	0.04	0.11		*		
Tp4_24549667^§^	0.19	0.00	*			
Tp4_24549680^§^	0.15	0.05	*			*
Tp4_26090577	0.16	0.12	**	*		
Tp4_27046602	0.09	0.08	**			
Tp5_3133717	0.21	0.02	*			
Tp5_9433815	0.21	0.15	*			
Tp5_10561679	0.17	0.06	***	*		
Tp5_11205553	0.09	0.09	*	*		*
Tp6_4397690	0.19	0.04	**			
Tp6_20345196	0.23	0.17	***	***	*	*
Tp7_28113385	0.17	0.07	**			
Tp7_28430847	0.10	0.05	*			
Tp7_28430875	0.10	0.05	*			


GBS of DNA from pooled leaf samples from eight populations (plots) generated 4556 SNPs. Hundred and fifty-six SNPs with variation in allele frequency among populations were identified with BayeScan. The SNPs for which allele frequencies were also significantly affected by stand type or seeding density according to analysis of variance are shown here. The 11 SNPs that were significant in a simple *F*_ST_-based test (*P* < 0.1, FDR 0.04) are indicated. ^*1*^Pairs of SNPs located on the same sequence tag are labeled with §. ^*2*^Significant effect in a two-way analysis of variance; *P < 0.05; **P < 0.01; ***P < 0.001.

### Chromosomal Regions of Identified SNPs Under Selection

The identified SNPs under selection were spread across all seven red clover chromosomes, and were in some cases closely or moderately linked (Figure 3). We took a closer look at the chromosomal regions around the SNPs with the largest allele frequency differences between populations (further details are found in Supplementary Tables S2, S4). The SNP with the largest allele frequency difference, a difference of 0.36 between Ps and Ms populations, was Tp4_11383593/11383594 located on chromosome 4. This SNP was not located in a known gene. There were also several SNPs located toward the distal end of chromosome 4. Among these, Tp4_23347367/23347286 and Tp4_24549667 had allele frequency differences between Ps and Ms populations of 0.28 and 0.19, respectively. The former one was located near an annexin and the latter one was located in an oxygenase and close to a transcription factor. The SNP with the second largest allele frequency difference between Ps and Ms (0.33) was Tp2_29283780 on chromosome 2. It was located in a stress-induced phosphoprotein and close to a syntaxin. Further up on chromosome 2 there was a region with many SNPs with moderate allele frequency differences between populations. Among these, Tp2_18474535, Tp2_18520944 and Tp2_18802245, were located in a WRKY family transcription factor, close to an LRR-like protein kinase, and in a RNA-binding protein, respectively. The SNP with the largest shift in allele frequency between the original population and the four survivor populations in study 1 was Tp3_5909984, with an average change in allele frequency of 0.22. This SNP was located in one of three adjacent membrane transport protein-like genes.

## Discussion

### Methodology

Several statistical methods have been developed to scan large numbers of loci across many individuals and link patterns of genetic variation to environmental variation (Holderegger et al., 2008; Schoville et al., 2012; Pannell and Fields, 2014). These methods identify outlier loci – loci with stronger differentiation in allele frequencies between populations than can be expected to occur due to random processes only, and which are, therefore, assumed to have been under selection. Statistically significant associations between genetic variation in outlier loci and variation in environmental variables indicate a role of the outlier loci in local adaptation. Adaptive outlier loci may represent new beneficial mutations that have increased in frequency and eventually become fixed in the population (hard sweeps). Alternatively, outlier loci represent alleles or haplotypes that have increased in frequency, but where some polymorphism is maintained (soft sweeps) (Barrett and Schluter, 2007). Soft sweeps can occur when selection on standing variation acts on multiple haplotypes in the genome simultaneously. Studies of local adaptation usually compare populations that have been exposed to contrasting conditions over many generations, and, in spite of migration, have evolved through repeated cycles of recombination and selection (e.g., Freeland et al., 2010; Turner et al., 2010; Gould et al., 2014). In some cases, such studies include replicates of populations that have started out from a common pool and been exposed to the same conditions; these replicates can be used to separate consistent signs of selection from random changes like genetic drift (Wiberg et al., 2017). The present study is different from these studies in the way that we characterize the selection (mortality/survival) that occurs *within* one generation only, with no reproduction or migration occurring. This allows for the use of a simple *F*_ST_-based test of changes in allele frequencies resulting from selection. We show that in spite of a large proportion of random mortality/survival, the use of several replicate survivor populations, sampled from replicate plots in a field experiment, improves the power of the test substantially, and makes it possible to remove these random effects and identify loci that have been under selection in all replicates. In study 1, BayeScan identified one of the SNP outliers identified by the simple *F*_ST_-based method, after combining the 3H and 5H replicates. In contrast, in study 3, where a higher number of individuals were pooled in each population sample and the differentiation between populations was larger than in study 1, BayeScan identified more potential outliers than the simple *F*_ST_-based method. Testing these SNPs further with analysis of variance made it possible to identify differential selection due to stand type and/or seeding density. In study 3, all outliers identified by the simple *F*_ST_-based method were included among those identified by BayeScan.

In order to be able to detect all loci with differences in allele frequency, it is necessary to have a sufficient coverage of the genome, i.e., a sufficiently high SNP density. A high SNP density can be achieved by using a restriction enzyme in the GBS protocol which is a frequent cutter (i.e., ApeK1, which we used), combining several restriction enzymes, and by sequencing to a sufficient read depth to be able to call SNPs and determine allele frequencies for the majority of restriction sites. The required SNP density depends on the linkage disequilibrium (LD) of the population. The lower the LD, the higher the SNP density needed in order for all genes to be in some degree of linkage with at least one nearby SNP. Red clover has a relatively small genome (approximately 420 Mb), facilitating good read depth relative to the sequencing effort, but varieties tend to have limited LD. The LD along the different chromosomes in the original population studied here has previously been characterized by De Vega et al. (2015), who found that the average LD, measured as R^2^, at distances of 100 Kb, ranged between 0.19 and 0.25 for the different chromosomes. At 500 Kb LD had decayed completely to background levels (R^2^ 0.02–0.05). The likelihood of detecting a locus with significantly different allele frequency in different populations depends on the magnitude of the allele frequency difference, the distance between the gene conferring the effect on survival and a linked SNP, and the LD in that specific region. Here, we obtained an average density of one SNP per 85 kb or 37 kb in study 1 and in study 3, respectively. The studied variety is a synthetic population with several possible haplotypes at any given chromosomal segment, thus all nearby SNPs might not necessarily be diagnostic, that is, distinguish between alleles with different effects on survival. Therefore, with the SNP densities obtained in our study, we are likely to pick up a substantial amount of loci affecting survival, but not all, particularly not in study 1.

Pooling of individual DNA samples, or of individual leaf samples prior to DNA extraction, can increase the allele frequency information obtained per sequencing effort, and allow for comparison of a large number of populations (Turner et al., 2010; Byrne et al., 2013; Wiberg et al., 2017). While sequencing of individuals requires a certain read depth in order to call SNPs and distinguish between homozygotes and heterozygotes, sequencing pools requires an even higher read depth for allele frequencies to be estimated accurately. Moreover, information about haplotypes and population structure is lost when sequencing pools. In our study, a very good correlation was obtained between allele frequencies obtained from a DNA pool of 88 individuals and allele frequencies obtained from genotyping of individuals (Figure 4 and Table 3). Read depth was increased only 7 times in the pool relative to the 88 individual samples (i.e., >10x reduction in sequencing effort), and a similar number of SNPs were obtained. At a MAF > 0.05 and a read depth in pools of 100-499, R^2^ was 0.97, while it was somewhat lower at lower and higher read depth. At the same MAF and read depth range, pooling of leaves of 100 plants *prior* to DNA extraction led to an average correlation of 0.87 and 0.90 in two sets of three replicates. This is slightly lower than that reported by Byrne et al. (2013), who obtained a correlation of *R* = 0.91 (*R*^2^ = 0.93) at MAF > 0.05 and read depth above 20x in replicate samples of leaves from around 200 perennial ryegrass seedlings. Pooling of individual leaf samples prior to DNA extraction reduces costs, but the accuracy of the allele frequency estimates is also reduced. Estimates could possibly have been improved if we had used more uniform leaf material and taken more care in sampling equal amounts of tissue from each individual. However, the use of several replicate populations compensates to some extent for the reduced accuracy of allele frequency estimates. The replicate samples from two of the populations in study 3 showed that there was considerable sampling error in our method. This could be overcome by sampling more individuals and/or by including replicate samples or populations in the study.

### Selection Occurring in the Field Within One Generation

The 88 plants in the original population sample represent the sown populations while the survivor populations represent subsets remaining in each plot after selection (survival) during 2.5 years of exposure to the prevailing field conditions and management. Such selection within one generation represents the environmental flexibility that the genetic variation within populations of outcrossing species can provide (Charles, 1964; Crossley and Bradshaw, 1968). Some alleles may contribute to yield in some environments, while other alleles contribute in other environments, making the population or cultivar robust to environmental variation. Our analyses of the genetic variation in the survivor populations as compared to the original population that was sown (study 1) showed that the survivor populations in four different plots had diverged from the original population in different directions. Thus, although the first PC-axis separated the two harvesting regimes (Figure 1), most of the allele frequency variation was random. This may reflect a response to unintended variation in the environment among plots, random selection of alleles at the majority of loci, or sampling error. The original population had only a very weak genetic structure, which remained in the survivor populations, indicating that there was no selection acting on the structure (Figure 2). In study 3, the first PC-axis separated Ps from Ms, and within Ps it separated the two seeding densities, suggesting that differential selection had occurred due to the different treatments (Figure 5).

If the original population has high genetic diversity and low LD (typical of forage cultivars), it cannot be expected that selection acting on a relatively limited number of loci will affect average genetic distance measured across the genome. In order to identify such selection, each individual locus must be considered. Indeed, by looking for allelic shifts of individual SNPs in several replicate survivor populations we identified loci that had been systematically selected under the prevailing conditions in the investigated field experiment (Figure 3). These are candidate loci for establishment success or persistence. In study 1, 12 SNPs, representing 11 loci, had significantly altered allele frequencies, measured as *F*_ST_, in Ps survivor populations (high seeding rate) relative to the original population. These SNPs represent loci with alleles conferring a higher likelihood for survival under the conditions that are common to all four plots. They may be related to, e.g., establishment, competition in Ps, winter survival and the general environmental and management conditions. The absolute average allele frequency changes detected ranged from 0.22 to 0.09. Tp3_5909984 was the SNP with the largest allele frequency shift from the original population to the survivor populations in study 1. It is located in the middle of the proximal half of Tp3. Interestingly, this is also the approximate location of the only QTL for persistence detected in a red clover mapping population of red clover by Herrmann et al. (2008).

In study 3, survivor populations were not compared with the originally sown population. Instead, survivors from Ps populations were compared to survivors from Ms populations, and survivors from populations sown at high seeding density was compared to survivors from populations sown at low density. A number of loci with allele frequencies indicating differential selection in Ps and Ms were identified. The absolute allele frequency changes detected were up to 0.36, suggesting that stand type exerted a relatively strong differential selection pressure. Red clover in mixture with perennial ryegrass and tall fescue experience earlier competition for light and possibly other resources, as the grasses grow and elongate earlier in the summer. Indeed, we have previously shown that offspring of survivor populations from Ms have earlier stem elongation than offspring from survivor populations from Ps (Ergon and Bakken, 2016), suggesting differential selection for earliness. Later in the summer, red clover plants are likely to experience stronger competition in Ps than in Ms, as individual red clover plants grow very large. Another condition that may vary between Ps and Ms is a stronger dependence of red clover plants on nitrogen fixation in Ms, as grasses have a more efficient nitrogen uptake and less is left for the clover.

Breeding, variety testing and seed multiplication of red clover occurs in Ps. Although seeding rates used usually are much lower (2–4 kg ha^-1^) than those in our experiment, our results suggest that unintended selection occurring in Ps during breeding and seed multiplication may not necessarily be in favor of good persistence in practical farming, were Ms are used.

## Conclusion

Making use of replicate populations and a simple *F*_ST_-based test, it was possible to identify loci that had been under selection within one generation in a red clover variety grown in a field experiment for two and a half years. Pooling of individual DNA samples or leaf samples before sequencing and estimation of allele frequencies reduce costs substantially, allowing analysis of multiple populations and treatments simultaneously. Sampling error must be controlled, e.g., by sampling a large number of individuals and/or sampling from several replicate populations. Characterization of genomic changes in survival experiments may be utilized in identification of genomic regions, genes and alleles conferring survival in red clover and other species under various environmental conditions, which again can be utilized in breeding. In addition to identifying loci associated with survival under the conditions prevailing in our field experiment, we have shown that there is differential selection occurring in pure stands of red clover as compared to red clover growing in species mixtures, suggesting that the use of pure stands in breeding might not identify the best genotypes for development of varieties to be used in species mixtures.

## Author Contributions

OR initiated the research. ÅE designed the experiments and wrote the manuscript. ÅE and VS conducted the experiments. ÅE, LS, and VS analyzed the data. All authors corrected and approved the final version.

## Conflict of Interest Statement

The authors declare that the research was conducted in the absence of any commercial or financial relationships that could be construed as a potential conflict of interest.
