# Supplementary material for: Allele Frequency Changes Provide Evidence for Selection and Identification of Candidate Loci for Survival in Red Clover (Trifolium pratense L.)
Source: Front Plant Sci. 2019 Jun 11;10:718. doi: 10.3389/fpls.2019.00718 (PMC6580991; doi:10.3389/fpls.2019.00718)
Supplement: Supplementary file 1 [file Data_Sheet_1.docx]

Supplementary Material

**Allele frequency changes provide evidence for selection and identification of candidate loci for survival in red clover (*Trifolium pratense* L.)**

**Åshild Ergon^*^, Leif Skøt, Vegard Eriksen Sæther, Odd Arne Rognli**

* **Correspondence:** Åshild Ergon: [ashild.ergon@nmbu.no](mailto:ashild.ergon@nmbu.no)

7.5 m^2^

**Figure S1**. Field experiment and sampling. Plots with the diploid red clover cultivar «Lea», both in pure stand (Ps) and in mixed stands (Ms) with white clover, perennial ryegrass and tall fescue, were included in an experiment sown in June 2010 at Ås, Norway (59⁰ 40’ N, 10⁰ 47’ E, 75 m a.s.l., annual precipitation 785 mm and average temperature 5.3 ⁰C). The experiment included two seeding rates (‘high’ (H) – 20 kg ha^-1^ and ‘low’ (L) – 10 kg ha^-1^) and two harvesting regimes (3 or 5 harvests per year, 3H and 5H). Leaves were sampled in October 2012, either as individual leaves (used in study 1, green plots), or as pools of single leaves from 100 individuals per plot (used in study 3, eight labelled plots in 3H). From the two Ps-H plots in 3H, three replicate leaf pools were sampled (used in study 3). In addition, leaves from individuals of the original population sown in the greenhouse were sampled (used in study 1 and 2).

**Table S1.** Number of SNP loci with a shift in allele frequency in all four red clover survivor populations relative to the original population at different significance levels, and with the corresponding false discovery rates (FDR) in study 1, using the simple F_ST_-based method. The 4966 SNPs with a minimum of 25 genotyped individuals in each of the five populations and a minimum minor allele frequency of 0.05 in the original population were tested.

| Significance level of chi square test for each survivor population | Number of SNP loci with a shift in allele frequency in all four survivor populations | Corresponding FDR |
| --- | --- | --- |
| P<0.1 | 27 | 0.018 |
| P<0.05 | 13 | 0.002 |
| P<0.01 | 0 | - |

**Table S2.** Mapping of sequence tags containing SNPs with a significant shift in allele frequency in four red clover survivor populations relative to the originally sown population (study 1), to the red clover genome (Tp2.0). SNPs were identified using a simple F_ST_-based method identified with the best hit for each sequence tag was selected. Seven sequence tags for which the hits had an E-value < 1 e^-18^, or only a partial alignment, are not listed.

| Sequence tag (position) | Chromosome/scaffold_position (Ref/Alt)^1^ | Predicted gene (position) | Near predicted genes in ± 5 kb region (position)^2^ | Synteny with other legume chromosomes^3^ |
| --- | --- | --- | --- | --- |
| TP21588 / TP103112^4^ (16031855-16031928) | Tp1_16031837 (G/***C***)  Tp1_16031875 (C/***G***) | Transmembrane protein, putative  (16031650-16032193) | - | Ca1 |
| TP73363  (18353318-18353381) | Tp1­_18353335 (***C***/T) | Trafficking protein particle complex subunit 9  (18352609-18355423) | Ribosomal RNA processing Brix domain protein (18348582-18351268)  Trafficking protein particle complex II-specific subunit 120 homolog (TRS120) (18356729-18359063) | Ca4 |
| TP146594  (24525073-24525136) | Tp1_24525118 (***C***/G) | ATP binding microtubule motor family protein  (24521687-24528526) | Unknown protein (24519319-24520271) | Mt4 |
| TP48637  (4384305-4384368) | Tp2_4384324 (***G***/A) | - | - | - |
| TP7660  (18474523-18474586) | Tp2_18474535 (A/***C***) | WRKY family transcription factor  (18469486-18477664) | - | Mt5, Ca2 |
| TP9013  (18802213-18802276) | Tp2_18802245 (T/***A***) | RNA-binding protein  (18799373-18804132) | Sulfite exporter TauE/SafE family protein (18806131-18815672)  Myb-like DNA-binding domain containing protein (18791298-18797862) | Mt1, Ca4 |
| TP24824  (5909914-5909971) | Tp3_5909984 (T/***C***) | Membrane transport protein-like  (5909372-5912799) | Membrane transport protein-like  (5906453-5909089)  Membrane transport protein-like  (5903780-5905702) | Mt3, Mt8, Ca5 |
| TP88042  (17626306-17626368) | Tp3_17626312 (***G***/A) | - | Phosphate transporter 4  (17624623-17625882)  Pentatricopeptide repeat (PPR) superfamily protein (17619833-17622757)  Ribonuclease 1 (17630373-17632677) | Mt5, Ca2 |
| TP120899  (571094-571154) | Tp4_571096 (C/***T***) | - | - | Mt5, Ca2 |
| TP17472  (19025642-19025705) | Tp4_19025686 (***T***/A) | C2H2-type zinc finger family protein | - | Mt2, Mt4, Ca1, Ca6 |
| TP98237  (20141734-20141797) | Tp4_20141773 (A/***G***) | - | Disease resistance protein, putative (20139740-20140484)  LRR and NB-ARC domain disease resistance protein (20143935-20155425)  Equilibrative nucleoside transporter 6 (20135605-20138352) | - |
| TP81268  (6458270-6458333) | Tp7_6458267 (***A***/G) | - | Ribosome-binding factor A family protein (6457308-6462752)  Amidophosphoribosyl-transferase  (6451690-6453062) | Mt3, Ca5 |
| TP11471  (23481175-23481238) | Tp7_23481186 (G/***A***) | - | Elongation factor Tu GTP-binding domain protein (23480307-23483179)  Sugar transporter 6 (23472875-23477699) | Mt2, Ca1 |
| TP47052  (135359-135422) | Scaff_537_135365 (T/***C***) | Histidine kinase-like ATPase  (132776-137370) | ATP/DNA-binding protein (137446-140239)  ATP/DNA-binding protein (126666-130848) | - |
| TP110520  (114138-114201) | Scaff_636_114141 (***G***/T) | - | Regulator of chromosome condensation (RCC1) family protein (107586-113165)  Alanine:glyoxylate aminotransferase 3 (117152-123334)  Alanine:glyoxylate aminotransferase 3 (116467-117105) | - |
| TP114603  (49039-49102) | Scaff_800_49089 (G/***C***) | - | ATP binding microtubule motor family protein (48264-51385) | Mt5, Ca8 |
| TP112120 (1733-1796) | Scaff_1608_1751 (***G***/A) | - | RNA-dependent RNA polymerase family protein (1620-8825) | - |
| TP129825 (909-970) | Scaff_22233_923 (A/***G***) | - | - | - |
| TP12160 (526-587) | Scaff_26753_588 (T/***C***) | Protein kinase superfamily protein (1-794) | - | - |

^1^ The major allele in the original population is underlined, the allele that has been selected is in bold italics. ^2^ SNPs had up to 14 additional genes located within ± 50 kb (see Table S2). ^3^ Ca, *Cicer arietinum*; Mt, *Medicago truncatula.* ^4^ Overlapping sequence tags.

**Table S3.** Number of SNP loci with a different allele frequency in four survivor populations from red clover pure stand plots relative to four survivor populations from species mixture plots, at different significance levels and with the corresponding false discovery rates (FDR) (study 3). The 4556 SNPs with a known chromosomal location, a minimum minor allele frequency of 0.05 and 100-499 reads in each of the eight populations were tested.

| Significance level of chi square test for each population pair | Number of SNPs with significantly different allele frequency (corresponding FDR) |
| --- | --- |
| 0.1 | 11 (0.04) |
| 0.05 | 6 (0.005) |
| 0.01 | 1 (0.00005) |
| 0.001 | 0 (-) |

**Table S4.** Position and surroundings of SNPs with a significant difference in allele frequency in survivor populations from pure stand plots vs. mixed stand plots (study 3) according to the simple F_ST_-based test.

| SNP locus (Ref/Alt) | Average allele frequency^1^ ± S.E. | | Absolute difference in allele frequency^2^ | Predicted gene (position) | Near predicted genes in ± 5 kb region (position) | Synteny with other legume chromosomes^3^ |
| --- | --- | --- | --- | --- | --- | --- |
|  | Pure stand populations (N=4) | Mixed stand populations (N=4) |  |  |  |  |
| Tp1_23903841 (G/A) | 0.19 ± 0.04 | 0.38 ± 0.04 | 0.19 * | Surface protease (23900940-23910097) | - | - |
| Tp2_7023442 (T/C) | 0.19 ± 0.02 | 0.36 ± 0.03 | 0.17 * | C2H2-like zinc finger protein  (7026504-7030401) | - | - |
| Tp2_18520944 (C/T) | 0.15 ± 0.01 | 0.34 ± 0.03 | 0.19 ** | - | Leucine-rich receptor-like protein kinase (18515555-18520583)  Pentatricopeptide repeat (PPR) superfamily protein (18521676-18524364) | Ca2, Mt5 |
| Tp2_29283780 (G/A) | 0.56 ± 0.04 | 0.24 ± 0.04 | 0.32 *** | Stress-induced phosphoprotein (29282793-29286735) | Syntaxin of plants 111 (29288553-29290970) | Ca8, Mt5 |
| Tp4_11383593 (G/A)  Tp4_11383594 (C/T) | 0.56 ± 0.09 | 0.20 ± 0.04 | 0.36 ** | - | Hypothetical protein (11384096-11395244) | Ca2, Mt5 |
| Tp4_23347267 (C/T)  Tp4_23347286 (A/G) | 0.42 ± 0.03 | 0.70 ± 0.04 | 0.28 ** | Unknown protein (23341476-23348173) | Annexin D8 (23351198-23355412) | Ca6, Mt8 |
| Tp4_24549667 (G/A) | 0.52 ± 0.01 | 0.71 ± 0.04 | 0.19 * | 2-oxoglutarate and Fe(II)-dependent oxygenase (24542963-24552049) | Transcription initiation factor TFIID, subunit TAF11  (24553718-24556649) | - |
| Tp5_10561679 (G/A) | 0.27 ± 0.03 | 0.11 ± 0.01 | 0.16 * | Unknown gene | Pentatricopeptide (PPR) repeat-containing protein (10552498-10557617) | Ca7, Ca8, Mt4, Mt5 |
| Tp6_4397690 (G/A) | 0.26 ± 0.02 | 0.46 ± 0.02 | 0.20 * | Unknown protein (4396830-4398590) | Mitochondrial transcription termination factor (4402323-4404880) | Ca3, Ca7, Mt7, Mt8 |

^1^ Frequency of the allele alternative to the reference genome. ^2^ The allele frequency for each mixed stand population was tested against the average of the pure stand populations and vice versa, and it was required that all eight F_ST_-values ( $\frac{\overline{q^{2}}-\overline{q}^{2}}{\overline{q}(1-\overline{q})}$) were significant; *, P < 0.1; **, P < 0.05; ***, P < 0.01; ****, P < 0.001. ^3^ Ca, *Cicer arietinum*; Mt, *Medicago truncatula*
